# Prevalence and distribution of Entamoeba species in a rural community in northern South Africa

**DOI:** 10.1016/j.fawpar.2020.e00076

**Published:** 2020-02-20

**Authors:** Amidou Samie, Leah Mahlaule, Peter Mbati, Tomoyoshi Nozaki, Ali ElBakri

**Affiliations:** a Molecular Parasitology and Opportunistic infections program, Department of Microbiology, University of Venda, Private Bag X5050, Thohoyandou, South Africa; b Botho University, Botho Education Park, Kgale, Gaborone, Botswana; c Department of Biomedical Chemistry, Graduate School of Medicine, The University of Tokyo, 7-3-1 Hongo, Bunkyo-ku, Tokyo, 113-0033, Japan; d Medical Laboratory Sciences Department, College of Health Sciences, University of Sharjah, Sharjah, United Arab Emirates

**Keywords:** Diarrhoea, *Entamoeba moshkovskii*, Rural, South Africa

## Abstract

Amoebiasis occurs worldwide and affects about 20–50 million people annually. Stool samples were collected from patients attending different rural clinics in Northern South Africa in the present study. Microscopic examination was performed for the initial detection of *Entamoeba* parasites. A multiplex PCR protocol based on the small subunit rRNA gene of *E. moshkovskii*, *E. dispar*, and *E. histolytica*, was used for the differential detection of the three *Entamoeba* species (collectively referred to as *Entamoeba* complex). A total of 170 participants were recruited in the study, with a mean age of 35.9 ± 17.8 years and a median of 37.0 years. The prevalence of *Entamoeba* species was found to be 34.7% and 33% by PCR and microscopy, respectively. *E. histolytica* had a prevalence of 4.1%, *E. dispar* 14.7% and *E. moshkovskii* 15.9%. Of the three species, only *E. histolytica* was significantly associated with diarrhoea and was more prevalent among HIV patients even in the absence of diarrhoea while the other two were not, although the difference was not significant (*p* > 0.05). This is the first study in South Africa to describe the prevalence of *E. moshkovskii*. *E. dispar* was significantly associated with abdominal pains (*p* = 0.003). Further studies are needed to clarify the role of *E. moshkovskii* and *E. dispar* in abdominal pain and diarrhoea.

## 1 Introduction

Amoebiasis is a common public health problem particularly in developing countries and is caused by *Entamoeba histolytica*. With the occurrence of two other non-pathogenic species of Entamoeba namely *E. dispar* (W.H.O., 1997) and *E. moshkovskii* (Ali et al., 2003), the pathogenic *E. histolytica* is often inaccurately reported or diagnosed. Although several studies have been conducted on the prevalence of *E. histolytica* around the world, most of the studies did not differentiate between *E. histolytica* and *E. dispar* due to the diagnostic methods used; resulting in an overestimation of the prevalence of *E. histolytica* (Kebede et al., 2003). Identified recently as a cause of infection in humans, *E. moshkovskii* endemicity is yet to be evaluated in many parts of the globe. Following its description, several studies have been conducted to determine its prevalence in human communities in different countries (Fotedar et al., 2008). In Iran, molecular studies showed that *E. dispar* was the predominant species especially in the central and northern areas of the country and that infection with *E. moshkovskii* may be common among Iranians while amoebiasis due to *E. histolytica* appear to be a rare infection in that country (Hooshyar et al., 2012).

Humans are the major host of *E. moshkovskii* and there does not appear to be other meaningful animal reservoirs of this parasite (Ximenez et al., 2011). Although previously thought to be a non-pathogenic organism, recent studies have shown that *E. moshkovskii* is capable of causing diarrhoea, colitis, and weight loss in mice (Hamano et al., 2006). Moreover, acquisition of *E. moshkovskii* infection was associated with diarrhoea among Bangladeshi children (Shimokawa et al., 2012) thus seemingly influencing the burden of amoebiasis. Previous studies in the Vhembe Districts in South Africa have indicated that the prevalence of *E. histolytica* in hospitals and among school children was 18.8% and 2.1%, respectively, whereas 25.3% and 8.5% of these study populations had *E. dispar*, respectively (Samie et al., 2006). However, this study did not include *E. moshkovskii*. Recently identified as a cause of infection in humans, *E. moshkovskii* endemicity has not been evaluated in most epidemiological studies. Apart from the report by Ngobeni et al. (2017) in low-income South African populations in Giyani and Pretoria where no *E. moshkovskii* was detected (0%), no other study has investigated its presence in the country. Hence, in the present study, we carefully determined the occurrence of the three *Entamoeba* species in the Vhembe and Mopani Districts of South Africa.

## 2 Materials and methods

### 2.1 Ethical considerations

The study was approved by the Health and Ethics committee of the University of Venda and the Limpopo Department of Health in Polokwane, South Africa. Approval was also received from the District Department of Health of the Mopani District as well as the Chiefs of the respective villages in the Limpopo province, northern South Africa. The objectives of the study were clearly explained to the participants, and those who agreed were asked to sign a consent form. Written informed consent was obtained from the parents or guardians of children prior to enrolment of the children in the research, and good clinical practice was followed.

### 2.2 Study participants' recruitment and data collection

Participants were recruited (February 2012 to September 2012) from Nkomo Clinic and Donald Fraser Hospital situated in the Mopani District and Vhembe District, respectively in the northern part of South Africa. Outpatients attending the clinics with intestinal complaints were recruited upon arrival at the clinics and asked to complete a questionnaire. The questions comprised of demographic information of the study participants, clinical and health related questions, socio economic status, and diarrhoeal history of the study participants. The children's mothers were asked to indicate if the child recently had diarrhoea and to specify the consistency of stools passed by the child in the past days. Anthropometric measurements including body weight, age and height were assessed and the Body mass index (BMI) was calculated for all participants as weight (in kilograms) divided by the square of height (in meters) and expressed as kg/m^2^.

### 2.3 Sample collection and microscopic examination

Stool samples were collected using a wide mouthed stool container and transported in cooler boxes filled with ice to the laboratory of Microbiology at the University of Venda. All specimens were properly labelled with patient's code, and date of collection. The specimens were transported to the laboratory within 4 h of passing of the stool, since amoebic trophozoites die and become unrecognizable after longer periods of time. Precautions were taken to prevent the samples from being contaminated with urine or dirt particles. Two wet mount slides (saline and Lugol's iodine) were prepared directly from each sample to increase the chance of detecting protozoan trophozoites and cysts as well as red and white blood cells. The smears were covered with cover slips and examined under the microscope using 40× objective lens. Cysts and trophozoites were identified as per their characteristic morphometric features.

### 2.4 DNA extraction from stool samples and PCR assay

Extraction of parasite genomic DNA from faecal specimens was done by the QIAamp DNA stool Mini kit from Qiagen (Qiagen, GmbH, Hilden, Germany), with some modifications following the beads beating in order to break down the cysts. About 0.25 g of glass beads were weighed and placed in an eppendorf tube containing 0.25 g of the stool sample. The tubes were placed in a bead beater and run for 1 min. After a brief vortexing, the protocol was continued with the Qiagen mini kit following the manufacturer's instructions. The PCR amplification reaction was performed in a final volume of 20 μl in a 0.2 ml PCR tubes by the use of a thermal cycler (G storm, Gene technologies, Braintree UK) following a previously described method (Hamzah et al., 2006). Reaction conditions were optimised to combine the forward primer (EntaF) with each of the three reverse primers (Ehr, Emr & Edr) in a single reaction mixture and under the same conditions.

The reaction mixture contained 0.2 μM of each forward and reverse primer, 10 μl dream Taq, a volume of 2.6 μl nuclease free water and 5 μl of extracted DNA samples. Amplification of each species-specific DNA fragment started with initial denaturation at 94 °C for 3 min, followed by 40 cycles of 94 °C for 1 min, 58 °C for 1 min, and extension at 72 °C for 1 min, with a final extension at 72 °C for 7 min. The samples were stored at 4 °C. Amplified products were visualised with ethidium bromide staining after electrophoresis on 1.5% agarose gel and a picture of the gel was taken using the gel documentation system from Genesnap.

### 2.5 Statistical analysis

The results of the study were analysed using the SPSS software, version 19. The chi square (χ^2^) test was used to determine the potential correlation between different demographic characteristics of the study participants who provided the samples and *Entamoeba* infections as well as other parameters such as clinical characteristics. The difference was considered significant when the *p* value was <0.05.

## 3 Results

### 3.1 Microscopic examination and PCR amplification

**Fig. 1:**
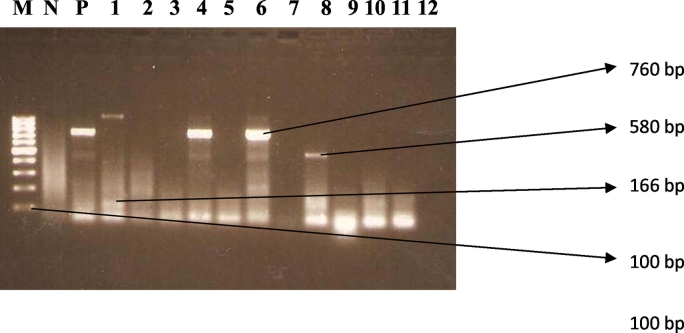
Representative images showing the agarose gel electrophoresis of the amplification of the *Entamoeba* DNA. The 166-, 580- and 760-bp bands depict specific amplification of DNA from *E. histolytica*, *E. moshkovskii,* or *E. dispar*, respectively. Lane “M” is the 100 bp ladder molecular weight maker. Lane “N” is no stool sample (negative control), P is positive control. Lanes 1–12 are representative clinical samples.

A total of 170 samples were collected from the same number of patients and microscopically examined by saline and iodine wet mount microscopy. Vegetative and/or cysts forms of *E. histolytica*, *E. dispar* and *E. moshkovskii* (collectively referred to as *Entamoeba* complex, as they are morphologically indistinguishable) were found in 57 (33%) samples. The extraction of DNA from the stool samples, followed by differential detection for the presence of *E. histolytica*, *E. dispar,* and *E. moshkovskii* by the PCR assay, with the use of species specific primers (EntaF and one of Emr, Edr, or Ehr) detected *Entamoeba* in 59 (34.7%) samples. The samples showed bands at 166 bp, 580 bp and/or 760 bp, which indicates *E. histolytica*, *E. moshkovskii,* or *E. dispar,* respectively (Fig. 1).

### 3.2 The prevalence of Entamoeba infections in the study population

A total of 170 samples were tested for *Entamoeba* by PCR. Of these, 27 (15.9%) samples were positive for *E. moshkovskii*, and 25 (14.7%) were positive for *E. dispar* and 7 (4.1%) were positive for *E. histolytica*. *E. histolytica and E. dispar* were found to be more prevalent in males (11.1% and 27.8% respectively) compared to females (5% and 19.8% respectively), while *E. moshkovskii* was more prevalent in the females (23.8%) compared to males (16.7%), but the difference was not statistically significant (*p* > 0.05). *E. moshkovskii* was more common in the age group 26–45 (27.5%), while *E. dispar* was more prevalent in the age group 0–25 with 23.3% occurrence and *E. histolytica* was more prevalent in the age group 26–45 with 7.5% occurrence than the other age groups, although the differences were not significant (*p* > 0.05). Individuals who were single had the highest prevalence of the parasites, *E. moshkovskii* (25.5%), *E. dispar* (25%) and *E. histolytica* (8.5%). All three *Entamoeba* species were more prevalent in patients who were not underweight compared to those who were [*E. moshkovskii* (6.9%), *E. dispar* (22.5%), and *E. histolytica* (23.5%)], although the difference was not significant (p > 0.05). *E. histolytica* was significantly more prevalent in the Vhembe district (*p* = 0.039). *E. moshkovskii* (27.3%) was significantly more prevalent in those who were not taking any medication at the moment, than those who were taking some form of medication (*p* < 0.05). The results are recorded in Table 1.

**Table 1:** The prevalence of *Entamoeba* infections in the study population as determined by PCR.

Characteristics of the population		*E. moshkovskii*	*E. dispar*	*E. histolytica*
Sex	Male	3(16.7%)	5(27,8%)	2(11,1%)
	Female	24 (23.8%)	20 (19.8%)	5 (5.0%)
	χ^2^	0.439	0.586	1.047
	p value	0.508	0.444	0.306
Age group	0–25	10 (23.3%)	10 (23.3%)	3 (7.0%)
	26–45	11(27.5%)	8(20%)	3 (7.5%)
	>45	6(16.7%)	7(19.4%)	1 (2.8%)
	χ^2^	1.280	0.208	
	p value	0.527	0.901	
Marital status	Divorced	0	0	0
	Married	4(20.9%)	13 (19.4%)	3(4.5)
	Single	12 (25.5%)	12 (25.5%)	4(8.5%)
	Widowed	1 (25.0%)	0	0
BMI	Not Underweight	7(6.9%)	23(22.5%)	24(23.5%)
	Underweight	0	2(11.8%)	3(17.6%)
	χ^2^	0.287	1.021	1.240
	p value	0.592	0.312	0.266
District	Mopani	27(65.9%)	25(65.8%)	7(46.7%)
	Vhembe	14(34.1%)	13(34.2%)	8(53.3%)
	χ^2^	0.442	0.413	4.265
	p value	0.506	0.520	0.039
Medication (drugs or antibiotics)	No	6(6.8%)	19(21.6%)	24(27.3%)
	Yes	1(3.2%)	6(19.4%)	3(9.7%)
	χ^2^	4.046	0.069	0.534
	p value	0.044	0.793	0.465

### 3.3 The demographic characteristics of the study population

A total of 132 of the 170 participants (77.5%) were females while 34 (20%) were males. Of the participants 89 (52.4%) were married, while 3 (1.8%) were divorced. The age of the study participants varied from 1 year to 76 years old. The mean age was 35.968 ± 17.81 years while the median age was 37.00 years. They were grouped into 3 age groups (0–25, 26–45, >45 years old), and most of the patients were aged between 26 and 45 years old (37.1%). Of the 170 participants, 72 (42.4%) indicated that they kept animals in their households. Of these 30 (41.7%) kept cattle, while only 4 (5.6%) had cats.

### 3.4 The occurrence of diarrhoea and related ailments among the study participants

Of the 170 study participants, 46 (27.1%) were taking antibiotics at the time of recruitment, and 32 (18.8%) had experienced some diarrhoea during the past 3 months. Of these 18 (56.3%) passed loose stools and 3 (9.3%) passed bloody stools. Of the 32 patients who experienced diarrhoea, 24 (75%) experienced acute diarrhoeal episodes while 8 (25%) had chronic diarrhoea. Abdominal pains were experienced by 79 (46.1%) of the study population. Of the 170 participants, 53 (31.2%) were coughing at the time of recruitment, and 39 (23%) indicated that they had difficulties in breathing. Among the study participants, 35 (20.6%) had some vision problems.

### 3.5 The health statuses of the study participants

Of the 170 participants, 22 (12.9%) were underweight according to Mchiza et al. (2015). A total of 58 (34.1%) reported to be HIV positive, of whom 30 (51.7%) were on anti-retroviral (ARV) treatment. Fourteen (8.2%) of the patients indicated that they had made use of alternative treatment, of whom 11 (78.6%) and 3 (21.4%) had received treatment from spiritual healers and traditional healers, respectively.

### 3.6 Relationship between the prevalence of Entamoeba infections and diarrhoea history of the patients

*E. moshkovskii* and *E. histolytica* were found to be more prevalent (25.2% and 11.5% respectively) in people who have a diarrhoea history than those who did not, while *E. dispar* was more prevalent in people without diarrhoea history (23.7%), although the difference was not significant (*p* > 0.05). *E. moshkovskii* and *E. dispar* were commonly found in watery stools (50% and 25% respectively) in patients who were experiencing diarrhoea, although the difference was not significant (*p* > 0.05). *E. histolytica* was more associated with mucous stools (50%) than the other types of stools, and the difference was significant (*p* < 0.05). *E. moshkovskii* was more associated with chronic diarrhoea (60%), than acute diarrhoea (9.5%), with a significant difference (p < 0.05). *E. dispar* and *E. histolytica* were associated more with acute diarrhoea than chronic, although the difference was not significant (*p* > 0.05). *E. dispar* was significantly associated with abdominal pains by 30.4% (*p* = 0.039). *E. histolytica* and *E. moshkovskii* were also associated with abdominal pains, but the difference was not significant (p > 0.05). The results are recorded in Table 2.

**Table 2:** The prevalence of *Entamoeba* infections according to history of diarrhoea among study participants.

Characteristics		*E. moshkovskii*	*E. dispar*	*E. histolytica*
Diarrhoea the last 3 months	No	22 (23.7%)	22(23.7%)	4 (4.3%)
	Yes	5 (25.2%)	3 (11.5%)	3(11.5%)
	χ^2^	0.227	1.798	1.922
	*p* value	0.634	0.180	0.166
Consistency	Bloody	0 (0%)	0(0%)	0(0%)
	Loose	3(18.8%)	2(12.5%)	1(6.3%)
	Mucous	0(0%)	0(0%)	2(50.0%)
	Watery	2(50.0%)	1(25.0%)	0(0%)
	χ^2^	3.653	2.725	14.861
	p value	0.455	0.605	0.005
Type of diarrhoea	Acute	2(9.5%)	2(9.5%)	2(9.5%)
	Chronic	3(60.0%)	1(20.0%)	1(20.0%)
	χ^2^	6.093	2.065	2.723
	p value	0.048	0.356	0.256
Abdominal pains	No	9(18.0%)	4(8.0%)	3(6.0%)
	Yes	18(26.1%)	21(30.4%)	4(5.8%)
	χ^2^	1.081	8.793	0.002
	p value	0.298	0.003	0.562

### 3.7 Relationship between the prevalence of Entamoeba infections and the clinical manifestations of the study participants

The prevalence of *E. moshkovskii and E. dispar* was high in the study participants who did not have any skin infections (25.5% and 21.6% respectively) as compared to those who did, although the difference was not significant (>0.05). *Entamoeba dispar* and *E. moshkovskii* were found to be more prevalent in the study participants who were currently having respiratory infections (71.4% and 57.1% respectively) than those who were not (*p* > 0.05). *E. histolytica* was significantly more prevalent in participants who were not coughing than those who were (*p* < 0.039). The prevalence of *E. moshkovskii* was higher in HIV negative participants (24.1%) than those who were positive (p > 0.05). *E. dispar* and *E. histolytica* were more prevalent in HIV positive patients (28.6% and 14.6%, respectively) than those who were HIV negative (*p* > 0.05).

### 3.8 Relationship between the prevalence of Entamoeba infections and animals in the households of the participants

Animals in the houses of the study participants were not associated with any infection, all *Entamoeba* species were found to be more prevalent in houses with no animals than those with animals, although the difference was not significant (*p* > 0.05). Donkeys were mostly associated with the presence of *E. moshkovskii* (33.3%) and *E. dispar* (33.3%), however the difference was not significant (p > 0.05). Goats were mostly associated with the presence of *E. histolytica* (8.3%), although the difference was not significant (*p* > 0.05).

### 3.9 Relationship between the prevalence of Entamoeba infections and the quality of drinking water used by participants

Drilled ground wells was the source of water which was most associated with the prevalence of *E. moshkovskii* (27%) *and E. histolytica* (6.3%) compared to all water sources, although the difference was not significant (*p* > 0.05). *E. moshkovskii*, *E. dispar*, and *E. histolytica* were found to be more prevalent in the patients who stored water for <3 days and the least affected were those who stored water for 3–4 days, although the difference was not significant (p > 0.05) (Table 3).

**Table 3:** Prevalence of *Entamoeba* infections according to drinking water sources and storage.

Characteristics		*E. moshkovskii*	*E. dispar*	*E. histolytica*
Source of drinking water	Borehole	17(27.0%)	12(19%)	4(6.3%)
	Communal tap	5(14.7%)	5(14.7%)	2(5.9%)
	Direct from the river	0(0%)	0(0%)	0(0%)
	Tap in the house	5(23.8%)	8(38.1%)	1(4.8%)
	χ^2^	2.206	4.920	0.135
	p value	0.531	0.178	0.987
Water storage	<3 days	6(24.0%)	6(24.0%)	2(8.0%)
	3–4	5(20.8%)	5(20.8%)	1(4.2%)
	>5	16(22.9%)	14(20.0%)	4(5.7%)
	χ^2^	0.073	0.178	0.334
	p value	0.964	0.915	0.846

### 3.10 Relationship between the socio-economic status of the patients and the prevalence of Entamoeba infections

*E. moshkovskii* was prevalent in patients who earned between R3001 (“R”, South African Rand; 1 Rand equal to 0.092 US Dollars as of on January 2014) and R4999, although the difference was not significant (*p* > 0.05). *E. histolytica* was highly prevalent in those with no dependents at all, although the difference was not significant (*p* > 0.05). Albeit all the educational levels were affected, *E. histolytica* and *E. dispar* were prevalent in those who had tertiary education (20% and 60% respectively), however the difference was not significant (p > 0.05) (Table 4).

**Table 4:** The prevalence of *Entamoeba* infections according to the socio-economic status of the study participants.

Characteristics		*E. moshkovskii*	*E. dispar*	*E. histolytica*
Income range	R1000	4(20.0%)	6(30.0%)	0(0%)
	R1001-R3000	10(17.2%)	10(17.2%)	5(8.6%)
	R3001-R4999	10(35.7%)	5(17.9%)	1(3.6%)
	R5000 & above	3(23.1%)	4(30.8%)	1(7.7%)
	χ^2^	3.773	2.384	2.383
	*p* value	0.287	0.497	0.497
Number of dependents	0	0(0%)	0(0%)	1(33.3%)
	1	6(20%)	4(13.3%)	1(3.3%)
	2–5	14(22.2%)	18(28.6%)	4(6.3%)
	6 & above	7(30.4%)	3(13.0%)	1(4.3%)
	χ^2^	1.799	4.914	4.558
	p value	0.615	0.178	0.207
Education	Illiterate	4(22.2%)	4(22.2%)	1(5.6%)
	Primary	2(18.2%)	4(36.4%)	1(9.1%)
	Secondary	20(23.5%)	14(16.5%)	4(4.7%)
	Tertiary	1(20.0%)	3(60%)	1(20%)
	χ^2^	0.184	7.214	2.221
	p value	0.980	0.065	0.528

## 4 Discussion

The present study indicated a high prevalence of *E. moshkovskii* compared to *E. dispar* and *E. histolytica* in stool samples from Mopani and Vhembe Districts in the Limpopo Province, South Africa (Samie et al., 2006). Microscopic examination of the samples showed a prevalence of 33% for all the *Entamoeba* species while PCR detected an overall prevalence of 34.7% of which most were *E. moshkovskii*. A similar study in Cote d' Ivoire reported a lower prevalence of *E. histolytica/dispar* (18.8%) among primary school children using microscopy (Ouattara et al., 2010). Unlike the present study, in Saudi Arabia a higher prevalence of *Entamoeba* complex (64.8%) was reported by microscopy among diarrheic samples revealing wide variation between different regions of the world (Al-Harthi and Jamjoom, 2007). Contrary to the Saudi study, the present study was not limited on diarrhoeal patients only but included general out-patients complaining of abdominal pains visiting the two clinics.

The results of the present study indicated that the recently discovered *E. moshkovskii* and the non-invasive *E. dispar* were more prevalent than the invasive *E. histolytica*. These findings are consistent with previous studies conducted on *Entamoeba* species (Pinheiro et al., 2004; Yakoob et al., 2012). It is important to point out that a much lower rate of infection with *E. histolytica* (4.8%) was observed in the present study than that in a previous study in the same region by Samie et al. (2006). This difference could be due to the type of population studied and the origin of the study participants. Most patients originated from Giyani while the previous study was conducted exclusively in the Vhembe region. In fact, the Mopani District represented by Giyani is dry with much higher numbers of diarrhoea compared to the Vhembe District (Samie et al., 2006).

The current study is the first to reveal the presence of *E. moshkovskii* (15.9%) in this region. A similar study in Yemen reported an *E. moshkovskii* prevalence of 18.2% (Al-Areeqi et al., 2017), while a higher frequency of *E. moshkovskii* (25.4%) was described from a rural area from Central Colombia (López et al., 2015). Interestingly, *E. moshkovskii* was found to be more prevalent than *E. histolytica* in our study.

Numerous studies reported the prevalence of *Entamoeba* complex among HIV+ and HIV−patients. Moran et al. (2005) reported a prevalence of 5.9% and 2.9% of *E. histolytica/dispar* among HIV+ and HIV− patients respectively using microscopy in Mexico City. On the other hand, higher prevalences of *E. histolytica* were detected (25.3% in HIV+ and 18.5% in HIV− patients) in the same Mexican study when the samples were retested by sensitive molecular tools (Moran et al., 2005). These results are consistent with ours which showed *E. dispar* and *E. histolytica* with a prevalence of 28.6% and 14.3% respectively in HIV+ patients and a prevalence of 20.5% and 5.4% respectively in HIV negative patients. These results are consistent with findings from our study. The prevalence of *E. dispar* and *E. histolytica* among HIV+ patients in the present report were 28.6% and 14.3%, respectively whereas in HIV negative patients a less prevalence was noted (20.5% and 5.4%, respectively). The prevalence of *E. moshkovskii* was higher in HIV negative participants (24.1%) than those who were positive (*p* > 0.05). Intriguingly, unlike a study by Al-Areeqi et al. (2017), neither age nor gender were significant risk factors for *E. moshkovskii* in the present report.

Similar to a study by Tasawar et al. (2010), no significant difference was noted in the present study between the prevalence of *E. histolytica* in males (11.1%) and females (5%). Moreover, no association between animal ownership and economic status of the participants and the prevalence of *Entamoeba* complex was observed. Previous studies in other countries have indicated that contact with animals and low personal hygiene are risk factors for infections with intestinal protozoa including *E. histolytica* (Alyousefi et al., 2011; Ismail et al., 2010). These results indicate the widespread occurrence of the cysts in the environment and the farms involved, which may serve as infective agents for the parasites in humans.

No significant difference was noted between the prevalence of *E. moshkovskii* and *E. dispar* in people with and without diarrhoea. However, the prevalence of *E. histolytica* was higher in patients who indicated that they had diarrhoea over the last three months. These findings are different from those obtained by Fadeyi et al. (2009), who found that *E. histolytica* and other intestinal parasites, were rare in acute and persistent diarrhoeic patients attending Ilorin hospitals by ELISA antigen detection method. The results of the current study are dissimilar to those obtained by Mojarad and colleagues in a study investigating the circulation of *E. moshkovskii* in patients with gastrointestinal disorders in Iran where a low prevalence (3.45%) was reported. On the other hand, like the current study the prevalence of *E. histolytica* (3.45%) was comparable to our result (Mojarad et al., 2010). In another study conducted in Turkey, *E. moshkovskii* was detected in two patients with diarrhoea and co-infected with *E. histolytica* (Tanyuksel et al., 2007).

Although *E. dispar* is known to be non-pathogenic, it was nevertheless significantly associated with abdominal pain (*p* = 0.003) in the present study, indicating the possibility that *E. dispar* might have some pathogenic characteristics, or these abdominal pains may be due to other causes. These results are similar to those obtained in a study conducted to determine the production of amoebic liver abscess by *E. dispar* xenic culture (Dolabella et al., 2012). In that study a culture of *E. dispar* strain (ICB-ADO) produced liver abscesses and tissue damage distinct from the frequently known non-pathogenic *E. dispar* (SAW 760) strain. These results are an indication that some *E. dispar* strains may be pathogenic. It is important to note that while no association existed between *Entamoeba* infections and the quality of drinking water used in the current study, sources of drinking water was a significant risk factor for *E. dispar* infection (Al-Areeqi et al., 2017).

This is the first study to identify *E. moshkovskii* in the region. Another African study reported an infection rate of 13% with *E. moshkovskii* in a cohort of HIV-suspected or confirmed inpatients from Tanzania (Beck et al., 2008). Moreover, this report found that *E. moshkovskii* was the most commonly species identified, yet it was not associated with neither diarrhoea nor abdominal pain. Unlike in the previous study conducted in the Vhembe District, the present survey showed a higher prevalence of *E. histolytica* in HIV positive patients even though the difference was not statistically significant. *E. histolytica* was more common among patients with diarrhoea. The present study also indicated that *E. dispar* was more common among participants who had abdominal pain. This may be an indication that *E. dispar* contributes to pathogenicity. However, further studies are needed using a larger study population in order to confirm this hypothesis.

## Declaration of competing interest

The authors declare that there are no financial and commercial conflicts of interest.
